# A 52-Week Real-Life Study of Baricitinib in Moderate to Severe Alopecia Areata: Clinical, Trichoscopic Assessment and Patient Reported Outcomes

**DOI:** 10.3390/jcm14228170

**Published:** 2025-11-18

**Authors:** Sara Lambiase, Danilo Cavalloro, Arnaldo Cioni, Enrico Matteini, Fabio Artosi, Francesca Poscente, Riccardo Belardi, Alessandro Terrinoni, Sergio Bernardini, Luca Bianchi, Elena Campione, Laura Diluvio

**Affiliations:** 1 Department of Dermatology, University Hospital of Tor Vergata, Viale Oxford 81, 00133 Rome, Italy; danilocavalloro@libero.it (D.C.); arnaldo.cioni@hotmail.com (A.C.); ematteini13@gmail.com (E.M.); fabio.artosi994@gmail.com (F.A.); fra.pos89@gmail.com (F.P.); luca.bianchi@uniroma2.it (L.B.); campioneelena@hotmail.com (E.C.); lauradiluvio@yahoo.it (L.D.); 2 Department of Experimental Medicine, University Hospital of Tor Vergata, 00133 Rome, Italy; belardiriccardo92@gmail.com (R.B.); alessandro.terrinoni@uniroma2.it (A.T.); bernards@uniroma2.it (S.B.)

**Keywords:** alopecia areata, baricitinib, trichoscopy, JAKi

## Abstract

**Background/Objectives:** Alopecia areata is an autoimmune condition characterized by rapid hair loss in the scalp, eyebrows, and eyelashes, for which treatments are limited. Baricitinib, an oral inhibitor of Janus kinases 1 and 2, has been recently approved to treat alopecia areata. **Methods:** A total of 23 patients affected by severe alopecia areata (SALT > 50) for more than 6 months were enrolled, including 14 with AU, 3 with AT, and 6 with ophiasis, and all of whom were treated with baricitinib for a minimum of 52 weeks. Clinical and trichoscopic assessments were performed at each visit, and the impact on quality of life, anxiety, and depression was evaluated using the Skindex-16 and the Hospital Anxiety and Depression Scale (HADS), respectively. **Results:** A total of 23 patients were enrolled, with a mean age of 44.62 years and a mean SALT of 83.66. The mean value of the SALT score decreased to 51.23 at 6 months and 42.41 after one year. Psychological well-being and quality of life also improved, as demonstrated by the decrease in Skindex-16 and HADS scores. Trichoscopic signs showed a decrease in yellow dots and black dots and an increase in vellus hairs and hair regrowth. Adverse events during the treatment period were reported in 18.75% patients. No drop-outs were registered. **Conclusions:** Data on the effectiveness and safety of baricitinib are promising and support the use of this drug in severe forms of AA, also in the early stages. We also suggest performing trichoscopy in response to therapy.

## 1. Introduction

Alopecia Areata (AA) is an autoimmune disorder, with an estimated lifetime risk of approximately 2.1% [1]. Although its exact etiology remains unclear, AA is thought to result from a combination of genes and environment. The condition is characterized by sudden, often unpredictable hair loss, which can affect individuals of various ages and backgrounds. AA manifests primarily as non-scarring hair loss, typically in well-defined patches on the scalp, but it can also affect other hair-bearing areas such as eyelashes and eyebrows. In severe cases, AA can progress to Alopecia Areata Totalis (AT), involving complete scalp hair loss, or Alopecia Areata Universalis (AU), which leads to total body hair loss. AA is associated with a significant psychological impact, often leading to depression, anxiety, and a reduced quality of life for affected individuals. The disorder is not merely a cosmetic concern but rather reflects a localized manifestation of a systemic autoimmune condition, frequently occurring alongside other autoimmune diseases. Thus, it is essential to consider AA as part of the broader context of autoimmune pathology rather than as an isolated dermatological issue.

The diagnosis of AA relies primarily on clinical evaluation, including a detailed patient history and assessment of comorbid conditions, alongside dermatoscopic examination. Characteristic findings, such as “exclamation point” hairs, black dots during acute phases, and yellow dots in chronic AA, are key diagnostic indicators. Laboratory tests are useful for excluding other autoimmune conditions, and histological analysis may reveal inflammatory cell clusters around hair follicles in a pattern known as “swarm of bee” lymphocyte infiltration.

Treatment strategies for AA are diverse and tailored to the individual patient’s clinical presentation, considering disease onset, duration, severity, and patient characteristics. Conventional therapies include topical corticosteroids, minoxidil, immunotherapy, and systemic treatments like corticosteroids, PDE-4 inhibitors, DMARDs, cyclosporine, and platelet-rich plasma [2]. Recent advances in the treatment of AA include Janus Kinase (JAK) inhibitors, with baricitinib emerging as a key therapy. Baricitinib, a first-generation JAK1 and JAK2 inhibitor, was originally developed for rheumatoid arthritis and later approved for AA. By inhibiting IFN-γ signaling in mouse models of AA and in patients with AA, baricitinib suppresses the inflammatory processes that lead to hair follicle damage. In fact, upon ligand binding to their specific cytokine receptors, Janus kinases (JAKs) become activated and phosphorylate signal transducers and activators of transcription (STAT) proteins. Once phosphorylated, STATs translocate to the nucleus, where they bind to defined DNA sequences and regulate gene transcription. JAK inhibitors (JAKi) can interfere with the signaling pathways of several cytokines involved in T cell-mediated inflammation and have demonstrated efficacy in various inflammatory and hematologic diseases [3,4,5].

Recent randomized controlled trials, including BRAVE-AA1 and BRAVE-AA2, have demonstrated that a 4 mg daily dose of baricitinib significantly improves hair regrowth in patients with severe AA, as evidenced by a higher proportion of patients achieving a Severity Alopecia Tool (SALT) score of 20 or less compared to those receiving a 2 mg dose or placebo [6]. In this context, a retrospective study conducted across 23 centers in Italy assessed the real-world effectiveness and tolerability of baricitinib in 118 adult patients with AA, providing further insights into its clinical utility and safety profile in daily practice. Our study aimed to evaluate the efficacy and safety of baricitinib in patients with severe AA. Specifically, we sought to assess its impact on hair regrowth, trichoscopic features, and patient-reported outcomes, including quality of life and psychological well-being, while monitoring for potential adverse effects and changes in biochemical and hematological parameters. Our results are consistent with both randomized trials and real-life studies on the use of baricitinib 4 mg for the treatment of moderate-to-severe AA.

## 2. Materials and Methods

This retrospective and prospective observational single-center study was conducted at the Dermatology Unit at Policlinico Tor Vergata, Rome, to assess the efficacy and safety of baricitinib in patients with AA. A total of 23 patients were enrolled, including 14 with AU, 3 with AT, and 6 with ophiasis, and all of whom were treated with baricitinib for a minimum of 52 weeks. The first aim of the study was to assess the efficacy of baricitinib in terms of hair regrowth, while the second aim focused on evaluating trichoscopic patterns before and during treatment. Inclusion criteria consisted of patients aged between 18 and 65 years, with a SALT score of 50 or higher, and an ongoing episode of AA lasting between 6 months and 8 years without spontaneous improvement in the past 6 months. Participants were required to have a minimum 6-month wash-out period from previous treatments, and all provided written informed consent. Exclusion criteria included pregnant or breastfeeding women, patients with malignancies, cardiovascular diseases, or severe liver dysfunction, as well as those with uncontrolled diabetes or who had received corticosteroids in the previous 8 weeks. Before starting treatment, all patients underwent a screening process, including a chest X-ray, ECG, and laboratory tests such as hepatitis and HIV serology, CPK, LDH, triglycerides, and QuantiFERON test. Patients were treated with baricitinib 4 mg once daily for 52 weeks, and clinical data were retrospectively analyzed at BL, W4, W12, W24, W36, and W52. Clinical parameters, including the SALT score, were evaluated at each visit, alongside patient-reported outcomes using the DLQI, Skindex-16, and Hospital Anxiety and Depression Scale (HADS), as well as biochemical markers such as liver enzymes, lipid profile, and complete blood count. SALT (Severity of Alopecia Tool) is a system used to measure the extent and severity of hair loss in patients with AA, which is an autoimmune condition that causes hair loss. Essentially, it helps doctors track how much hair is lost from the scalp. The score ranges from 0 to 100. A score of 0 means there’s no hair loss, while 100 means complete hair loss on the scalp. The higher the score, the more severe the hair loss. SALT is really helpful for monitoring how a patient’s condition progresses and how effective treatments are. For example, if a patient’s SALT score decreases after starting a treatment, that usually means the treatment is working. SKINDEX (Skin Index) is a tool used to measure the impact of a skin condition on a person’s quality of life. It’s especially useful for conditions like AA, psoriasis, eczema, and other dermatological issues. The scale includes a questionnaire that asks patients about how their skin condition affects different areas of their lives. The Skindex is divided into three key areas: functional impact: how the condition affects daily activities; emotional impact; how the condition affects feelings, mood, and emotions; and cosmetic impact: how the condition impacts a person’s appearance and self-esteem. It helps doctors understand not just the physical effects of the condition, but also how it affects the patient’s emotional well-being and everyday life. This makes it easier to tailor treatment approaches that consider both the body and the mind. HADS (Hospital Anxiety and Depression Scale) is a psychological assessment tool that is commonly used in hospital settings to measure the levels of anxiety and depression in patients. It’s particularly useful because it focuses on identifying symptoms of these conditions without being influenced by physical illness or medications. The scale is made up of two main parts: anxiety (HADS-A): questions about how anxious, worried, or nervous the patient feels; depression (HADS-D): questions about feelings of sadness, hopelessness, or disinterest in life. Since anxiety and depression are common in patients with chronic conditions like AA, the HADS can help ensure patients get the mental health support they need in addition to treatment for their physical symptoms.

Dermoscopic evaluations were performed at each visit to evaluate trichoscopic features such as yellow dots, black dots, dystrophic hairs, exclamation point hairs, and vellus hairs.

Statistical analysis was carried out by collecting and organizing demographic and clinical data in an internal database, with quantitative variables expressed as means and qualitative variables as percentages. Differences between scores at different time points were analyzed using repeated measures ANOVA followed by Bonferroni’s post-hoc test, with statistical significance set at *p* < 0.05. This study design provided a comprehensive evaluation of the effects of baricitinib in patients with AA, focusing on both clinical outcomes and safety parameters across a 52-week treatment period.

## 3. Results

**Figure 1:**
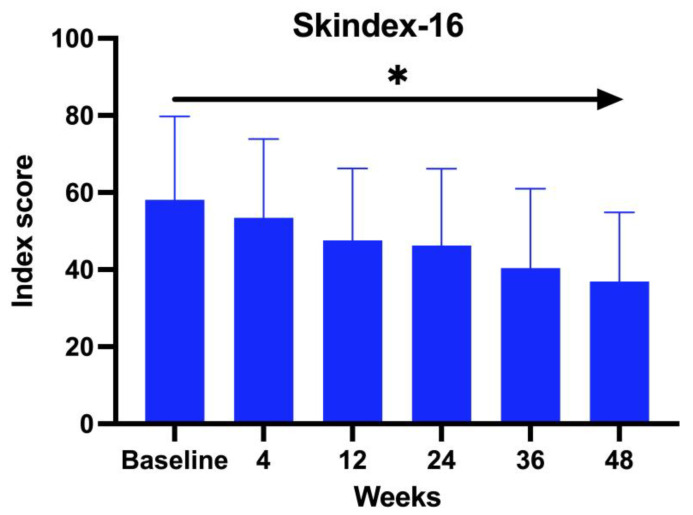
Skindex-16 score. * The ANOVA test was statistically significant with *p* < 0.05.

A total of 23 patients with a mean age of 44.62 years (range 19–64 years old), comprising 5 males (22%) and 18 females (78%), were treated with baricitinib for a period of at least 52 weeks. Regarding family history, 63.64% of the patients claimed to have a familiar predisposition to autoimmune diseases. The average duration of AA was 22 years (range 2–52 years). 61% were affected by AU, 13% by AT, and 26% by ophiasis. Patients’ comorbidities were recorded: 78.26% of the patients enrolled in the study presented with an underlying disease in concomitance with AA. Among these, 37% were affected by Hashimoto thyroiditis, 16% psoriasis, 16% hypercholesterolemia, 11% diabetes, 10% blood hypertension, and, finally, 5% glaucoma and 5% undifferentiated connectivitis. All the 23 patients enrolled in the study underwent a different line of previous treatment: 38% were treated with topical corticosteroids, whereas 36% with systemic corticosteroids, 14% topical immunotherapy, 10% cyclosporine, and 2% methotrexate. We evaluated the DLQI questionnaire, Skindex-16, at BL and subsequently at each visit. The Skindex-16 questionnaire showed a mean value at BL of 56.23 and then decreased at each visit, with an average of 51.48 after 1 month (W4), 46.66 at W12, 45.21 at W24, 37.52 at W36, and 36.53 at W52 (ANOVA; *p* < 0.026). (Figure 1)

**Figure 2:**
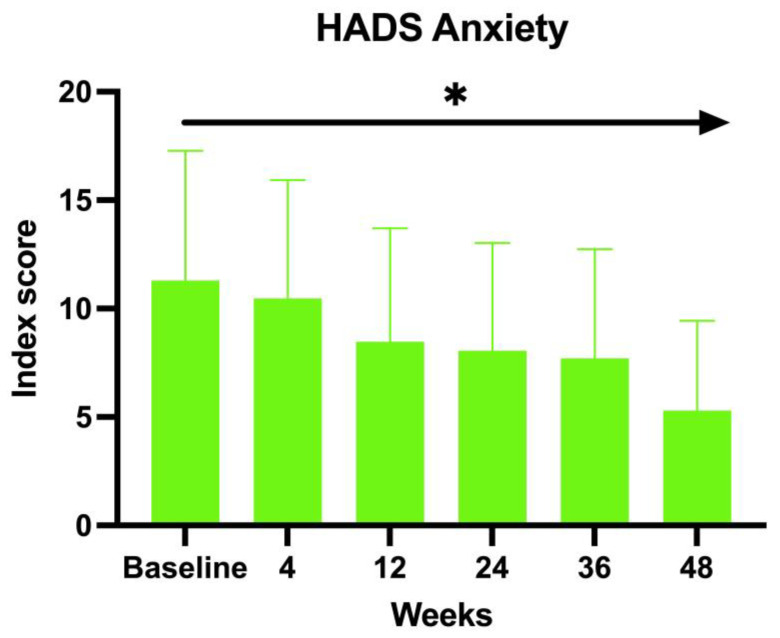
HADS anxiety score. * The ANOVA test was statistically significant with *p* < 0.05.

The enrolled patients were also asked to fill in the HADS. Both the anxiety and depression scores showed an inverse proportion to the duration of the treatment with baricitinib. The baseline HADS Anxiety value at BL was 12.83, 11.88 at W4, 9.97 at W12, 10.04 at W24, 9.04 at W36, and 8 at W52 (ANOVA; *p* < 0.01) (Figure 2).

**Figure 3:**
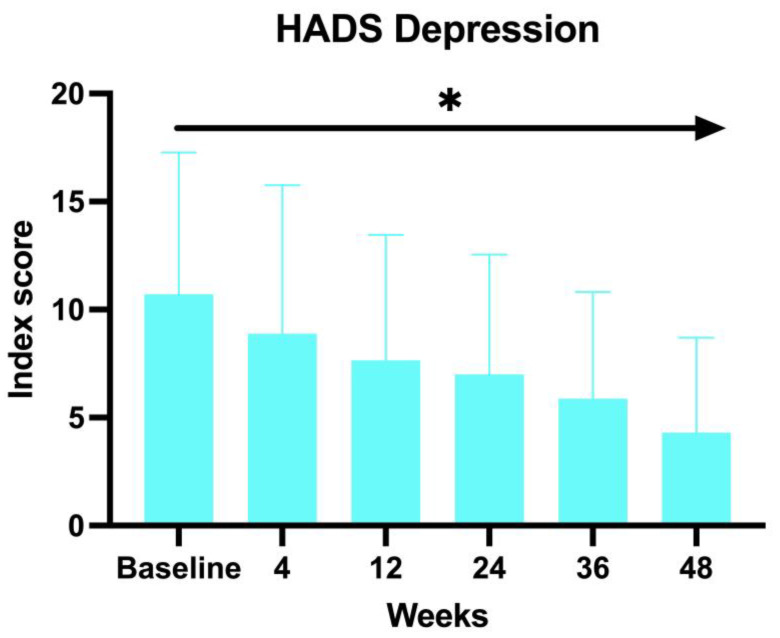
HADS depression score. * The ANOVA test was statistically significant with *p* < 0.05.

A similar decrease has been observed with the HADS Depression score, with an average of 12.66 at BL, 11.64 at W4, 9.64 at W12, 9.76 at W24, 8 at W36, and 7 at W52 (ANOVA; *p* < 0.03) (Figure 3).

**Figure 4:**
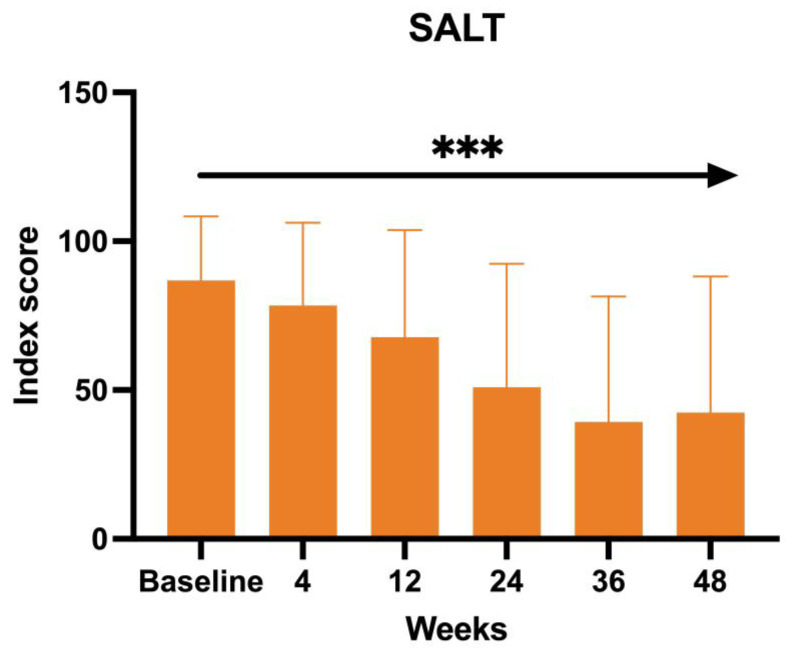
SALT score. *** The ANOVA test was statistically significant with *p* < 0.001.

The SALT score, directly proportional to the efficacy of the treatment of baricitinib in terms of hair growth, has also been measured at each visit. The mean value at BL was 83.66, 76 at W4, 64.71 at W12, 51.23 at W24, and 37.77 at W36, with a final value of 42.41 at W52 (ANOVA; *p* < 0.0005) (Figure 4).

A trichoscopic evaluation was performed during each visit. The five characteristics we assessed included yellow dots, black dots, dystrophic hair, exclamation mark hair, and vellus hair. At BL, the most prominent features were black dots and yellow dots, with all the patients presenting this characteristic (100%). The second most common feature was exclamation marks with 20.93%, followed by dystrophic hairs (11.62%). After one month (W4), we observed a decrease of black dots (60%), yellow dots (84.44%), and exclamation marks (6.66%). A complete absence of dystrophic hairs and an increase of vellus hairs (35.55%). After three months of treatment, the trend observed during the previous visit continued: with yellow dots (75.60%) and black dots (24.39%). No dystrophic hair was found. The exclamation marks were observed in 2.43%, and the vellus hair had an increase of 43.90%. After six months, the features found were as follows: yellow dots 73.52%, black dots 29.41%, exclamation marks 2.94%, absence of dystrophic hairs and vellus hairs in 52.94%. After nine months of treatment: yellow dots were found in 71.42%, black dots in 23.8%. No variation in the percentage of exclamation marks and dystrophic hairs compared to six months of treatment. Hair regrowth occurred in 71.42% of patients enrolled. Finally, after 1 year of treatment, we observed yellow dots in 52.94%, black dots in 11.76%. No dystrophic hair and exclamation marks were found. Vellus hairs in 41.17% of patients. The test used to perform the statistical analysis was the Student’s *t*-test (*p* < 0.05). Regarding the different biochemical markers, we did not observe statistically significant changes. The development of adverse events has been reported in 18.75% of the enrolled patients: three patients (13%) complained genital candidiasis, two patients (8%) had menstrual irregularities, followed by Herpes simplex reactivation (4%), low-grade squamous intraepithelial lesion (LSIL) due to the reactivation of the HPV virus (4%), urinary tract infection (4%), benign breast lesion (4%) and 4% complained an event of pre-syncope.

## 4. Discussion

AA is a complex dermatological autoimmune condition characterized by the sudden loss of hair in several patterns [1]. It is a multifactorial disease involving genetic factors and environmental factors, such as stress, smoking, diet, sleep habits, and alcohol consumption [7]. The disease has an important effect on the psychological well-being of the patients, representing both a trigger in the development of Alopecia and a consequence of the disease. The patients affected by AA often claim a decrease in their quality of life, associated with psychosocial challenges, including feelings of depression, diminished self-esteem, changes in self-image, and a reduction in the frequency and enjoyment of social interactions [8,9]. The severity of AA can be divided into several categories, with the most common: AA patchy, AT, AU, and AA ophiasis [4]. These clinical variants range in severity and are generally assessed with the SALT score [10]. Predicting the regrowth of hair in individuals with hair loss is challenging, as several factors act as negative predictive signs. These include persistent skin lesions lasting over a year, early onset of hair loss before puberty, a family history of hair loss, the presence of a distinctive “ophiasis” pattern in hair loss, nail abnormalities, coexisting autoimmune diseases, and the occurrence of Down syndrome. Notably, AT and AU subtypes tend to have less favorable outcomes. In a comprehensive 2019 review of six studies involving 375 AT and AU patients, only 8.5% achieved complete hair regrowth (32 out of 375). Moreover, other studies indicate that complete recovery from these subtypes occurs in fewer than 10% of cases. One of the new JAK-STAT inhibitors, baricitinib, was approved in 2022 for the treatment of AA. Baricitinib has a high affinity for JAK1 and JAK2 and inhibits their activity, preventing STAT phosphorylation and activation [11]. The use of baricitinib for treating AA was investigated in two phase 3 trials, BRAVE-AA1 and BRAVE-AA2, involving adult patients with severe AA. The BRAVE-AA1 and BRAVE-AA2 studies examined the effects of baricitinib (4 mg, 2 mg) versus placebo in patients with severe AA. Patients receiving 4 mg of baricitinib showed the best outcomes, with over 30% achieving a SALT score ≤ 20 by week 36, and 39% achieving it by week 52. Additionally, more than 31% in the 4 mg group had significant improvements in eyebrow and eyelash hair loss. The 2 mg group also showed improvement, though to a lesser extent. Both doses led to better hair loss scores and were associated with improvement of AA [6].

**Figure 5:**
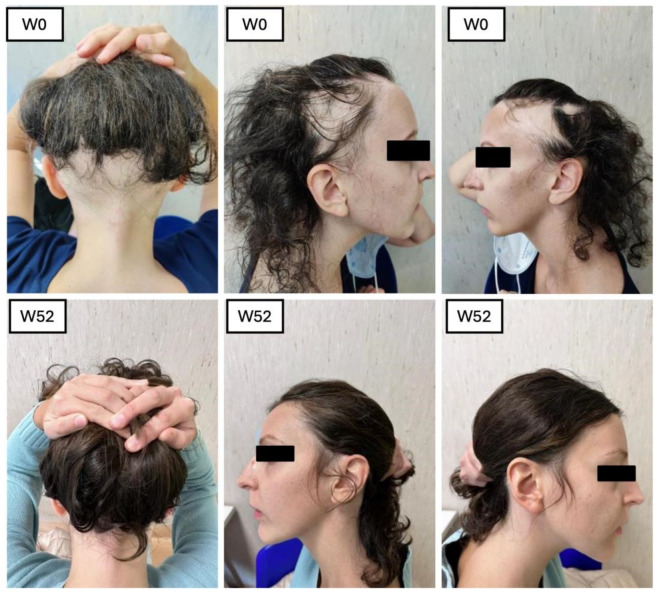
Patient 1 clinical improvement in SALT score.

**Figure 6:**
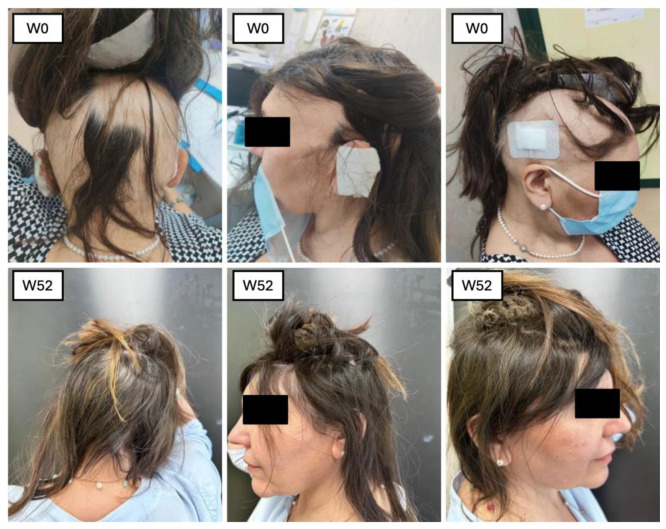
Patient 2 clinical improvement in SALT score.

Our study had a multifaceted objective. At its core, we sought to determine the efficacy of baricitinib concerning its ability to facilitate hair regrowth in individuals with severe AA. In addition, we placed a strong emphasis on ensuring the safety of this treatment by carefully monitoring and assessing any adverse effects during its administration. Furthermore, our study delved into trichoscopic characteristics [12]. This dual approach allowed us to comprehensively evaluate the treatment’s impact on both hair regrowth and the well-being of the individuals undergoing the treatment. Our study involved a sample of 23 patients who underwent treatment with baricitinib for at least 52 weeks. All these patients were affected by severe AA, with the mean SALT score at BL 83.66. The efficacy of the drugs was confirmed by the reduction in clinimetric indexes as the SALT score, in patients’ reported outcomes through Skindex-16 and HADS questionnaires, and an improvement in hair growth was noted in all the patients at trichoscopic evaluation. In terms of hair regrowth, both SALT and trichoscopy showed a significant improvement. The SALT score showed a decrease at each visit, from W4 to W52 (Figure 5 and Figure 6).

The second clinimetric score was measured through trichoscopy. At baseline, the most common features were black dots and yellow dots (100%). These two features progressively decreased with the duration of the treatment, and, finally, after 52 weeks, the most prominent characteristics observed were vellus hairs and hair regrowth. Simultaneously, the yellow dots dropped to 52.94% and the black dots to 11.76%. There were no dystrophic hairs or exclamation marks. This information further enforces the evidence of baricitinib’s efficacy in the treatment of AA. Both clinimetric scores were statistically significant (ANOVA; *p* < 0.05). In terms of patient-reported outcomes, two quality-of-life impact assessment questionnaires were given to the patients at each visit. The Skindex-16 questionnaire showed a mean value at baseline of 56.23 and then decreased at each visit, with 51.48 after 1 month (W4), 46.66 after 3 months (W12), 45.21 after 6 months (W24), 37.52 after nine months (W36), and 36.53 after one year (W52). Both the anxiety and depression scores showed an inverse proportion to the duration of the treatment with baricitinib. The baseline HADS Anxiety value was 12.83, and was 8 at W52. A similar decrease has been observed with the HADS depression score, with a mean value at baseline of 12.66 and 7 at W52. This data further enforces the efficacy of baricitinib in the improvement of quality of life, underlining not only its benefit on hair growth, but also on the psychological aspect of the patients. Both patient-reported outcome scores were statistically significant (ANOVA; *p* < 0.05). In our study we analyzed the variation of the biochemical markers, such as of HDL, LDL and total cholesterol, triglycerides, glycemia, leukocytes, and creatinine before the start of the treatment and at each visit, but none of the biochemical markers variations measured were statistically significant (ANOVA; *p* > 0.05), confirming the safety of baricitinib in terms of hematological assessment. The safety of baricitinib was also examined in terms of the development of adverse effects. A total of 18.75% of the patients participating in the study experienced some adverse effects during the treatment: 13% genital candidiasis, 8% menstrual irregularities, 4% Herpes simplex reactivation, 4% low-grade squamous intraepithelial lesion (LSIL) due to the reactivation of the HPV virus, 4% urinary tract infection, 4% benign breast lesion and 4% complained an event of pre-syncope.

## 5. Conclusions

AA is a multifactorial autoimmune condition that significantly impacts patients’ psychological well-being and quality of life. Predicting outcomes in AA is challenging. Traditional treatment options have shown limited success, leading to the exploration of innovative approaches. Baricitinib’s approval in 2022 for AA has opened new possibilities for patients. Our study shows encouraging results, with significant improvements in hair regrowth, quality of life, and psychological well-being. Despite some non-specific side effects reported by a portion of patients, the safety profile of baricitinib appeared favorable. However, larger studies are needed to establish any definitive associations between these side effects and the treatment. Although the study had limitations related to sample size and follow-up duration, the positive outcomes observed underscore the potential of baricitinib as a promising therapeutic option for individuals with severe AA. Furthermore, interindividual differences in immunological background may play a critical role in influencing both disease progression and response to treatment.

## Data Availability

Aggregate data available on request.

## Abbreviations

The following abbreviations are used in this manuscript:

AA	Alopecia areata
AT	Alopecia totalis
AU	Alopecia universalis
JAK	Janus kinase
SALT	Severity Alopecia Tool
BL	Baseline
HADS-A/D	Hospital Anxiety and Depression Scale—Anxiety/Depression
SKINDEX	Skin index
LSIL	Low-grade squamous intraepithelial lesion
